# Efficacy of Percutaneous Transarterial Embolization in Patients with Spontaneous Abdominal Wall Hematoma and Comparison between Blind and Targeted Embolization Approaches

**DOI:** 10.3390/jcm11051270

**Published:** 2022-02-25

**Authors:** Stefano Di Pietro, Francesco Tiralongo, Carla Maria Desiderio, Francesco Vacirca, Stefano Palmucci, Francesco Giurazza, Massimo Venturini, Antonio Basile

**Affiliations:** 1 Radiology Unit 1, Department of Medical Surgical Sciences and Advanced Technologies “GF Ingrassia”, University Hospital Policlinico “G. Rodolico-San Marco”, University of Catania, 95123 Catania, Italy; stefanodp1608@gmail.com (S.D.P.); carladesiderio@hotmail.it (C.M.D.); f.va77@libero.it (F.V.); spalmucci@unict.it (S.P.); basile.antonello73@gmail.com (A.B.); 2 Interventional Radiology Department, Cardarelli Hospital of Naples, 80131 Naples, Italy; francesco.giurazza@aocardarelli.it; 3 Department of Diagnostic and Interventional Radiology, Circolo Hospital, Insubria University, 21100 Varese, Italy; massimo.venturini@uninsubria.it

**Keywords:** embolization, radiology, interventional, angiography, digital subtraction, hematoma, abdominal muscles

## Abstract

Background: Endovascular treatment of abdominal wall hematomas (AWHs) has been increasingly used when conservative treatments were not sufficiently effective, and it is often preferred to surgical interventions. The aim of our study was to evaluate the safety and technical and clinical success of percutaneous transarterial treatment of AWH and to evaluate the efficacy of blind embolization compared to targeted embolization. Materials and Methods: We retrospectively enrolled 43 patients (23 men and 20 females) with spontaneous AWH who underwent digital subtraction angiography (DSA) and embolization, focusing on the presence of signs of bleeding at pre-procedural CT-Angiography (CTA) and at DSA. Furthermore, we divided patients into two groups depending on blind or targeted embolization approaches. Results: The mean age of the study population was 71 ± 12 years. CTA revealed signs of active bleeding in 31 patients (72%). DSA showed signs of active bleeding in 34 patients (79%). In nine patients (21%), blind embolization was performed. The overall technical success rate was 100%. Clinical success was achieved in 33 patients (77%), while 10 patients (23%) rebled within 96 h, and all of them were re-treated. No major peri-procedural complication was reported. The comparison between blind and targeted embolization showed no statistically significant differences for characteristics of groups and for clinical success rates (78% and 77%, respectively, −*p* = 0.71). The technical success was 100% in both groups. Conclusions: Our study confirms that transarterial embolization is a safe and effective option for the treatment of spontaneous AWHs, and it suggests that the efficacy and safety of blind embolization is comparable to non-blind.

## 1. Introduction

Abdominal wall hematomas (AWHs) are defined as the extravasation of blood in muscle structures that can be contained by the fascia or can diffuse into the peritoneal or retroperitoneal space. AWHs are relatively uncommon findings, but they may be a life-threatening condition, often associated with other conditions such as abdominal trauma, anticoagulation or iatrogenic injuries. The exact incidence and prevalence of AWH, however, are still unknown [1,2,3,4].

Traumatic AWHs are correlated with blunt or non-blunt injuries involving the abdomen; their detection is important for their strict association with potentially severe visceral and vascular injuries [5]. Iatrogenic vascular injuries of the abdominal wall muscles can occur after open surgery, laparoscopy, intra-abdominal injections, paracentesis or peritoneal catheter insertion [3].

Anticoagulant and antiplatelet therapies are the most common causes of spontaneous abdominal wall hematomas, but spontaneous bleeding may also occur in patients with coagulation disorders or damage to blood vessels, such as hypertension, atherosclerosis, vasculitis and hematologic disorders [6].

The knowledge of the vascular and topographic anatomy of the abdominal wall allow the mechanism of injury and the likelihood of hemorrhagic onset in defined regions of the abdominal wall to be understood.

**Figure 1:**
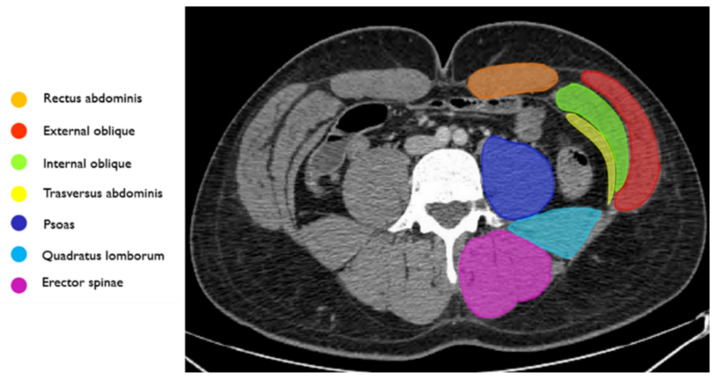
Annotated axial CT image of the abdominal wall musculature anatomy.

The abdominal wall is divided into anterior, lateral and posterior compartments. There are four anterior abdominal wall muscles per side: the rectus, the external oblique, the internal oblique and the transverse muscle (Figure 1).

**Figure 2:**
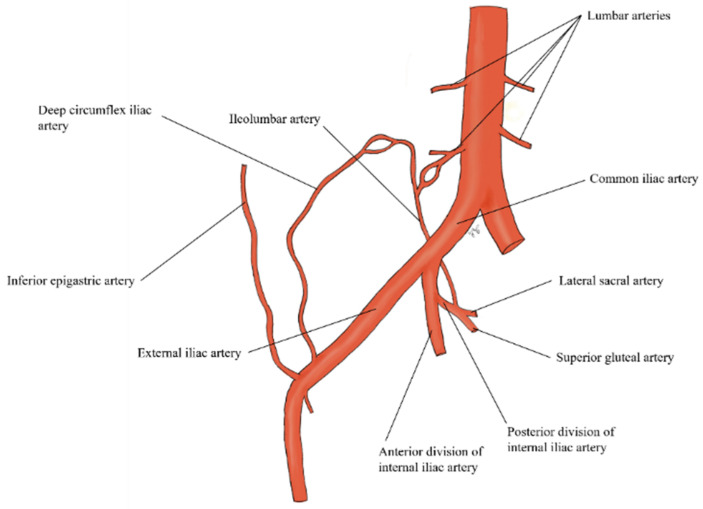
Drawing shows the arterial supply of anterior and posterior abdominal wall muscles.

The vascular supply of these muscles is mainly provided by the deep inferior epigastric arteries (IEA), the superior epigastric arteries (SEA) and the deep circumflex iliac arteries (CIA). The IEA reach the posterior aspect of the rectus muscle where they join the SEA coming from the internal thoracic arteries. The posterior muscles are three per side: the iliac, the psoas and the erector spinae muscles. These muscles are supplied by the lumbar arteries (LA) and the iliolumbar arteries (ILA). The ILA is the first ascending branch of the posterior gluteal trunk of the internal iliac artery [6] (Figure 2).

AWHs of the anterior wall most often occur in the lower quadrants of the abdomen, due to the anatomic characteristics of this region. Above the arcuate line of Douglas (a horizontal or transverse line that lies just below the umbilicus), in fact, rectus muscles are fully enclosed both anteriorly and posteriorly by the fascial sheath that arises from the aponeuroses of the external oblique, internal oblique and transversalis muscles. This sheath protects both the superior epigastric artery and the inferior epigastric artery. Below the arcuate line, the sheath is present only anteriorly, and the epigastric vessels are placed between the transversalis fascia and the peritoneum. For these reasons, hematomas that occur above the arcuate ligament are most often contained, small and secondary to distal lesions, while hematomas located below the arcuate line of Douglas are generally bigger, can be secondary to proximal lesions and can cross the median line and sometimes reach near the posterior peritoneum and the retropubic space [6,7].

The diagnosis of AWHs, especially in case of posterior wall hematoma, is challenging due to heterogeneous clinical presentations, and imaging techniques are usually required. Computed tomography (CT) is essential to provide accurate information about location, size, origin, and extension of hematomas but also to exclude other acute abdominal conditions, while CT-Angiography (CTA) has a crucial role to demonstrate the presence of active bleeding within the hematoma [8].

Depending on the patient’s condition, the underlying cause and the extent of the bleeding, the management of the AWHs can include conservative, surgical or endovascular treatment [3].

Management of AWH remains controversial and relies currently on conservative measures such as correction of coagulation parameters, fluid repletion and blood transfusion [9]. In hemodynamically stable patients, the common management currently continues to be conservative. It was suggested that more aggressive approaches should be reserved for unresponsive and complicated patients [10]. In case of hemodynamically unstable patients, surgical intervention such as open surgery with exploration and ligation of the bleeding vessel is taken into account, but this technique often fails [2,4]. In recent years, catheter-directed angiography and embolization have become additional options for patients with life-threatening AWH [2].

Endovascular treatment of the abdominal wall hematomas consists in transarterial embolization of the target vessels identified by CTA or digital subtraction angiography (DSA) using different materials such as coils, absorbable sponge or glue or a combination of these materials, and this technique is nowadays considered a fast and efficient treatment option [11]. In the absence of proof of extravasation on DSA, a blind or empiric embolization of the suspected vessel based on topographic reference and findings of CT examination can be performed [12].

The aim of our study was to evaluate spontaneous abdominal wall hematomas, focusing on the detection of active bleeding at CTA and DSA imaging and on the safety, technical and clinical success of percutaneous endovascular treatment and to evaluate the efficacy of blind embolization compared to targeted embolization, where active bleeding was demonstrated at DSA.

## 2. Materials and Methods

### 2.1. Study Population and Setting

We retrospectively analyzed the files from our RIS (Radiology Information System) and PACS (Picture Archiving and Communication System) of all consecutive patients with abdominal wall hematoma who underwent emergency angiographic evaluation and embolization between January 2018 and November 2021.

The initial search retrieved a total of 49 patients. Patients who recently underwent surgical intervention (*n* = 3) or had recent trauma (*n* = 3) within the 3 weeks before the diagnosis of AWH were excluded because they were considered to have potentially traumatic or iatrogenic hematoma. Overall, the study population included 43 patients.

Clinical and laboratory data such as pre- and post-interventional hemoglobin and International Normalized Ratio (INR) were obtained from the digital medical records.

### 2.2. Pre-Interventional CTA

All patients underwent CTA before DSA. The CT examinations were performed using two different scanners: Optima 660 (GE Healthcare, Chicago, IL, USA) and Toshiba Aquilon Prime (Toshiba Medical Systems Corporation, Otawara, Japan).

The CT protocol consisted of a non-contrast phase, followed by an arterial phase (acquired with an automatic bolus tracking technique, with the ROI (Region of Interest) placed in the proximal abdominal aorta), a venous phase (acquired with a delay of 60 s) and a delayed phase (acquired with a delay of 120 s).

A bolus of 80–120 mL of Iomeprol (Iomeron 350 or 400 mg/mL; Bracco Imaging, Milan, Italy), followed by 30 mL saline flush, was administrated using an automated contrast injection system at a flow rate of 4–5 mL/s. The CT images were reformatted in both coronal and sagittal planes. The location of the hematoma was classified as an anterior or posterior abdominal wall hematoma. Signs of active bleeding (i.e., active extravasation of the contrast medium and pseudoaneurysm formation) were evaluated. In patients without signs of active bleeding at CTA, it was decided to proceed with DSA evaluation and eventual embolization because of a patient’s unstable hemoglobin levels or because of the continuous need of transfusion to maintain acceptable hemoglobin levels (>8.0 g/dL).

### 2.3. Diagnostic Angiography and Transarterial Embolization

DSA and TAE were performed by interventional radiologists with at least 5 years of experience and by radiology residents attending a third or fourth year of training. DSA and endovascular treatments were performed using a Toshiba Infinix-i CAS-880A Cath Angio Lab (Toshiba Medical Systems Corporation, Otawara, Japan).

After skin disinfection and sterile draping, a 10-cm long 5 Fr guiding sheath was introduced via the right (preferred) or left common femoral artery. Arteriography was performed with Iomeprol (Iomeron 350, Bracco Imaging, Milan, Italy) as a contrast agent, with a frame rate between two to six images per second.

In patients whose pre-interventional CTA did not show active bleeding or the target vessel was not clearly identified, overview angiograms with a 4 or 5 Fr catheter placed in the abdominal aorta were performed. In all other cases, CTA-guided selective arteriography of the most likely bleeding site was immediately performed. Selective catheterization was performed with the use of 4 or 5 Fr appropriate diagnostic catheters, such as Cobra 2 (Cordis, Santa Clara, CA, USA) or Sos-Omni (AngioDynamics, Latham, NY, USA), and an angled, flexible 0.035-inch hydrophilic guidewire (Radiofocus; Terumo, Tokyo, Japan). Super selective or distal catheterization was usually conducted with a 2.7 Fr preloaded microcatheter system, which was inserted coaxially into the diagnostic catheter.

**Figure 3:**
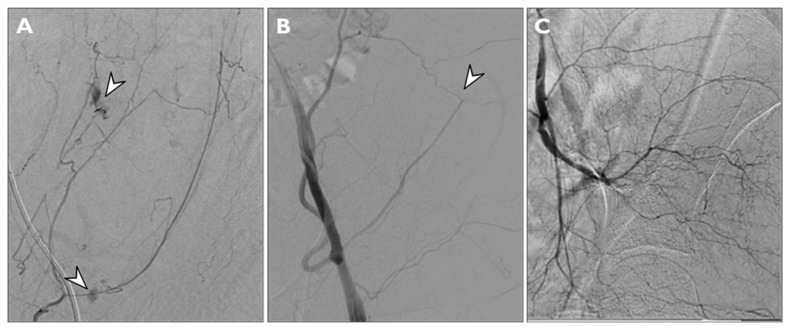
Findings considered for angiographic proof of bleeding: direct signs of active bleeding—contrast blush extravasation (arrowhead in (**A**)), indirect signs—cut-off vessel sign (arrowhead in (**B**)) and absence of active bleeding (**C**).

Findings considered for angiographic proof of bleeding were direct signs of active bleeding (contrast blush extravasation, focal spot of enhancement, hemorrhagic petechiae) indirect signs (vessel cut-off sign), pseudoaneurysm formation and the absence of active bleeding (Figure 3).

Embolization was carried out via the microcatheter using pushable or detachable coils (deployed coil diameter: 2–6 mm), a temporary embolic agent such as a re-absorbable gelatin sponge (Spongostan^®^, Johnson & Johnson Medical N.V., New Brunswick, NJ, USA), a combination of coils and gelatin sponge or a Squidperi 18 (Emboflu, Gland, Switzerland). The choice between the embolic materials was determined by the discretion of the treating interventional radiologist and considering the vascular anatomy and the position of the microcatheter within the target vessel. The microcatheter was placed as close as possible to the bleeding site to release the embolic material, and after embolization, the adjacent vessels were catheterized selectively to exclude the presence of collateral vascular flow to the bleeding source. At the end of the procedure, the guiding sheath was left in place in case of a recurrent bleeding for 24–48 h. Other data considered for our study were the target vessel location, the procedural timing (more or less than 60 min) and the time between pre-procedural CTA and angiography.

### 2.4. Laboratory Findings

The following laboratory values were recorded: pre- and post-interventional hemoglobin and the pre-interventional International Normalized Ratio (INR). The pre-interventional values were gathered at a maximum of 12 h before the embolization procedure. The corresponding post-interventional values were gathered within 24 h after the procedure.

### 2.5. Definitions

Targeted embolization was defined as the embolization of vessels where direct or indirect signs of active bleeding were demonstrated on the DSA study.

Blind embolization was defined as the embolization of a target vessel without angiographic proof of extravasation, typically guided by CTA findings in normal-appearing vessels [13]. Technical success was defined as the complete embolization of all target vessels.

Clinical success was defined as an absence of clinical, laboratory or radiological signs of re-bleeding within a 96-h window after procedure. The 96-h follow-up was based on clinical and laboratory parameters and, when indicated, CTA evaluation. Clinical failure was considered in patients who presented signs of re-bleeding (hemoglobin decrease, hypovolemic shock or evidence of persistent bleeding at post-procedural CTA examination) and who needed to undergo a new angiographic procedure followed by embolization of bleeding vessels. Minor and major procedure-related complications were defined according to the Society of Interventional Radiology [14].

### 2.6. Blind Versus Targeted Embolization

Furthermore, we divided all patients into two groups depending on the embolization approach, blind versus targeted embolization, to analyze the differences between the two study populations and to compare clinical and technical success rates and the rate of complications between these groups and these approaches.

### 2.7. Statistics

A statistical analysis was performed using the MedCalc program (MedCalc version 11.4.4.0, MedCalc Software bvba, Mariakerke, Belgium). Continuous variables are presented as the mean ± SD. Categorical variables are reported as percentages. Characteristics of the study population are reported as the mean (SD), range and median. For comparative statistics, a chi-square test was performed between the blind and targeted embolization groups’ gender, clinical success rates and rates of complications.

The comparison between the mean values of age, pre-procedural hemoglobin and INR and delay between CT and DSA was assessed using an independent samples *t*-test. A *p* value of >0.05 was considered as non-statistically significant.

## 3. Results

### 3.1. Study Population and Laboratory Findings

The initial search retrieved a total of 49 patients. After exclusion of patients with potentially traumatic or iatrogenic hematoma, 43 patients (53% of males, mean age of 71 ± 12 years) with spontaneous AWH were enrolled in the study (Figure 3). The mean value of pre-procedural hemoglobin in our population was 8.51 ± 1.87 g/dL. The mean pre-procedural INR was 1.25 ± 0.26. The mean post-procedural hemoglobin was 8.78 ± 1.36 g/dL. The characteristics of study population are summarized in Table 1.

### 3.2. Pre-Interventional CTA

CTA revealed signs of active bleeding within the hematoma in 31 patients (72%). In 12 patients (28%), CTA did not show signs of active bleeding. In 15 patients (35%), the hematoma was localized within the anterior or lateral abdominal wall (rectus abdominis, external oblique, internal oblique and transversus abdominis muscles) and in the posterior abdominal wall (iliopsoas, psoas and glutei muscles) in 28 cases (65%) (Table 2).

### 3.3. Diagnostic Angiography and PTAE

Angiography and TAE were performed with a mean delay of 7 h and 50 min (range 1 h and 14 min–22 h and 30 min) from CT.

DSA showed direct signs of active bleeding in 32 patients (74%) and indirect signs of bleeding (cut-off vessel sign) in 2 cases (5%). In 9 patients (21%), no angiographic evidence of active bleeding was found (Table 2). TAE was performed in one arterial territory in 25 (58%) patients, in two arterial territories in 13 (30%) patients, in three arterial territories in 2 (5%) patients and in four arterial territories in 3 (7%) patients.

The embolized arteries were the epigastric inferior artery (*n* = 17), lumbar artery (*n* = 21), iliolumbar artery (*n* = 17), deep circumflex iliac artery (*n* = 7), hypogastric artery (*n* = 2), deep femoral artery (*n* = 1), phrenic artery (*n* = 1) and intercostal artery (*n* = 2).

Overall, a total of 69 arteries were embolized, corresponding to an average of 1.4 arteries per patient. Embolization materials used were absorbable gelatin foam (Spongostan^®^; Johnson and Johnson, New Brunswick, NJ, USA) in 15 (35%) patients, coils in 5 (12%) patients, a combination of gelfoam and coils in 22 (51%) patients and a liquid embolic agent (Squidperi^®^; Emboflu, Gland, Switzerland) in one case (2%). Blind embolization was performed in 9 patients out of 43 (21%). The mean time for the angiographic procedure was less than 60 min in 53% of the procedures, exceeding 60 min in 47%.

The technical success rate was 100%. Clinical success was achieved in 33 patients (77%) with no need for further interventions. Ten patients (23%) had evidence of recurrent bleeding within a 96 h-time window, and all of them were re-treated with a new TAE procedure: 5 patients had a recurrence within 24 h from DSA, one patient re-bled after 48 h, 2 patients rebled after 72 h and 2 patients had a longer gap with re-bleeding at 96 h. It is worthwhile to mention that one patient who had been re-treated with TAE for rebleeding within 24 h from DS, needed and underwent a third TAE procedure because he showed signs of re-bleeding 48 h later.

No major peri-procedural complication was reported. Minor complications were seen in only one patient (for a resulting overall rate of complications of 2%), who presented a hematoma of the femoral vascular access site, demonstrated with angiography, and that was treated with manual compression at the end of the procedure (Table 3).

### 3.4. Blind Versus Targeted Embolization

The characteristics of the study populations of the two groups who underwent blind and targeted embolization are summarized in Table 4. The comparison between the two groups did not show statistically significant differences for gender, mean age, mean pre-procedural hemoglobin and INR, localization of AWHs and mean delay between CT and DSA. Technical success was obtained in 100% of patients in both groups. Clinical success rates were 77% for the group who underwent targeted embolization and 78% for the group who underwent blind embolization; 2 out of 9 (22%) patients treated with blind embolization and 8 out of 34 (23%) patients treated with targeted embolization rebleed after the first TAE. The chi-squared test showed a value of 0.13 (*p*-value = 0.7180), showing no statistical significance between the clinical success rates of AWHs patients treated with blind and targeted embolization (Table 4).

## 4. Discussion

Abdominal wall hematomas, involving the anterior or posterior abdominal wall, are relatively rare clinical conditions. The incidence of this condition is not clear due to the lack of focused studies, but some authors have reported that rectus sheath hematoma occurs only in about 1.8% of patients presenting with acute abdominal pain [15], while hematomas of the psoas muscle occur in about 0.1–0.6% of patients presenting with risk factors such as elder age, undergoing hemodialysis and anticoagulation therapy [16,17].

In general, the AWH management choice is guided by the patient’s condition, and it could be conservative or invasive. Since most hematomas are self-limiting, the first-line approach is usually conservative, consisting of volume support, reverse anticoagulation, analgesia, management of eventual risk factors, compression of hematoma and ice treatment. Invasive treatments, such as surgery or transarterial embolization of the bleeding vessels, are currently reserved to patients with expanding hematomas, that are hemodynamically unstable or with neurological impairment (especially in posterior wall hematomas). In recent years, the endovascular treatment of AWH has been increasingly used for patients in which conservative treatments were not sufficiently effective, and it is often preferred to surgical interventions [2,3].

According to the literature, AWH is a condition more commonly observed in elderly patients, especially those with comorbidities [18]. In a study conducted by Klausenitz et al. [2] on 30 patients who underwent DSA and embolization therapy for retroperitoneal bleeding, the mean age of the patients was 71.9 ± 9.8 years; in a review of the literature on rectus sheath hematoma, Hatjipetrou et al. [3] revealed a mean age of patients suffering from this condition ranging between 46 to 69 years. Moreover, a study performed by Barral et al. [19] on 112 patients with spontaneous soft-tissue hematomas who underwent TAE revealed a mean age of 72 ± 14 years. Our data, with a mean age of patients of 71 ± 12 years, are consistent with the findings of the literature.

**Figure 4:**
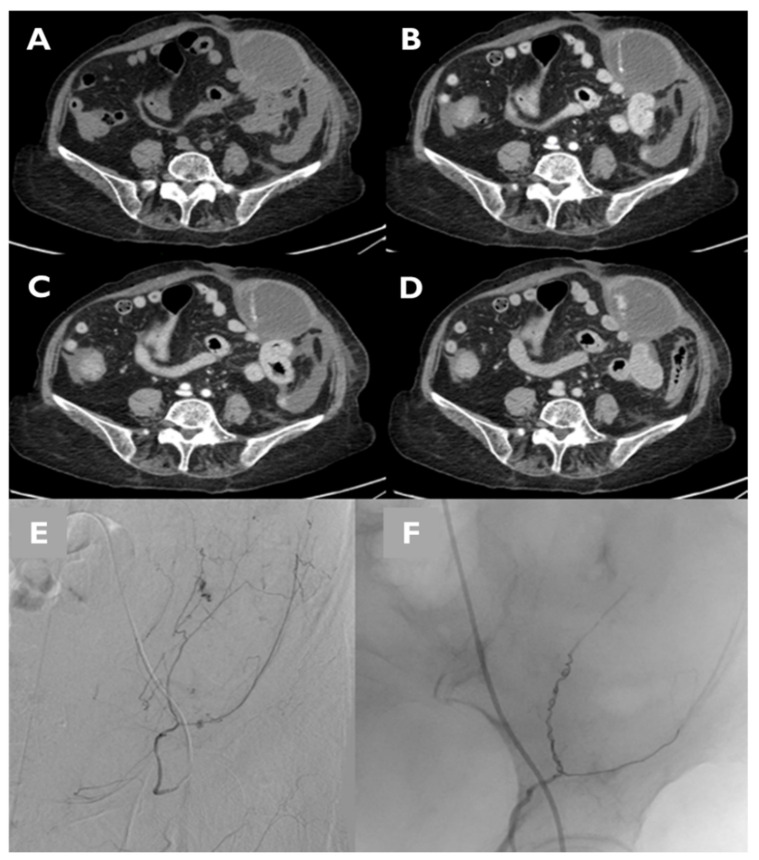
CT and DSA images of a patient presenting with hypovolemic shock. Evidence of a large hematoma within rectus abdominis below the arcuate line in axial CT images. The pre-contrast phase shows the extension and location of the hematoma (**A**). The contrast-enhanced acquisition at the arterial (**B**), portal (**C**) and delayed phase (**D**) allow the active bleeding to be detected. Angiography of the same patient shows contrast blush from branches of the inferior epigastric artery (**E**). TAE was performed using 3-mm coils and a Gelatin sponge (**F**).

**Figure 5:**
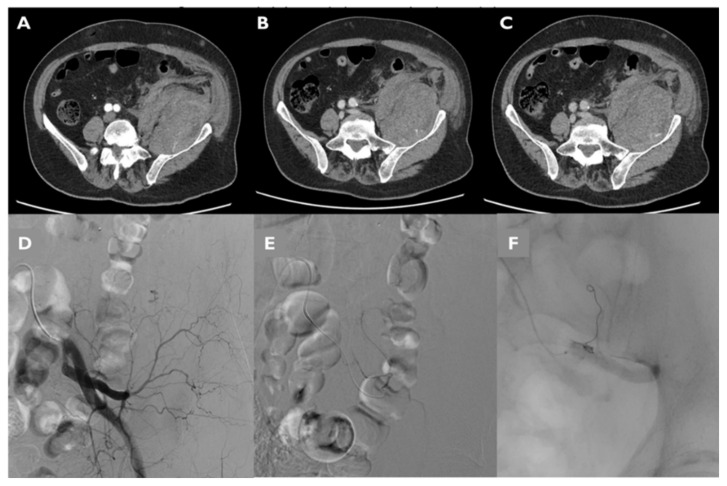
CT and DSA images of a patient presenting with a large hematoma of the iliopsoas muscle extending into the retroperitoneal space. Tri-phase contrast-enhanced axial CT images show active bleeding in the arterial (**A**), portal (**B**) and delayed phase (**C**). DSA shows active extravasation from left ILA (**D**). After super selective catheterization (**E**), PTAE was performed using a 3-mm coil and a gelatin sponge (**F**).

CT angiography has gained a key role in the pre-procedural setting to identify signs of active bleeding, identifying the bleeding vessel and to help interventional radiologists plan angiography and embolization procedures [6] (Figure 4 and Figure 5). In our study, CTA demonstrated active bleeding in 72% of patients, a rate higher than the findings of Touma et al. [12], who reported a detection rate of active bleeding of 47%, but lower than the findings of Klausenitz et al. [2], who had shown a rate of 93%. The experience of Barral et al. [19], instead, reported a rate of 88% of active bleeding visibility at CT imaging. In addition, our data on the detection of active bleeding on DSA (rate of 79%) are consistent with the literature, since Touma et al., Klausenitz et al. and Barral et al. have reported a rate of, respectively, 85%, 73% and 70% [2,12,19].

Blind embolization is often used for endovascular treatment of gastrointestinal bleeding when active bleeding is not identified on DSA, but, to our knowledge, currently there is a lack of published studies that compare the technical and clinical outcomes of this approach versus non-blind embolization in AWH patients. However, in literature, some cases of empiric embolization in patients with AWH without active bleeding have been sporadically reported. A systematic review performed by Touma et al. regarding the TAE of spontaneous soft tissue hematomas (concerning abdominal wall musculature), collected 267 patients out of 63 studies, and 37 of those patients (13.8%) underwent empiric (or blind) embolization [12]. In our experience, instead, 9 out of 49 patients (21%) were treated with blind embolization. This could suggest that, in our center, a quite aggressive approach was used in the treatment and interventional strategies of AWH.

Regarding the bleeding vessels identified at DSA and that have been subsequently embolized, in our experience, the most affected vessels were the lumbar and iliolumbar arteries (21 and 17 times, respectively) for posterior abdominal wall hematomas and the epigastric inferior artery (17 times) for anterior abdominal wall hematomas. These findings are in line with the result of Klausenitz et al. [2], who reported that 81.8% of all the bleeding vessels in posterior AWH were the lumbar and iliolumbar arteries, and with the results of Rimola et al. [20], where the epigastric inferior artery was the primary source of bleeding in all 12 patients with rectus sheath hematoma.

In 18 cases out of 43 (42%), TAE was performed in more than one arterial territory, and we observed that 15 of these 18 cases were patients with posterior AWH. Moreover, in 5 of these 18 cases, TAE was performed in 3 or 4 arterial territories, and 4 of these 5 cases were patients with posterior AWH. These findings suggest that posterior AWH could be more complex to treat, because more frequently there could be the need to embolize more vessels compared to anterior AWH, although definitive conclusions cannot be made, and this hypothesis should be considered cautiously.

In line with other experiences [12,20], even in ours, the embolic material most often used was a combination of gelfoam and coils, while Klausenitz et al. [2] used NBCA alone in the majority of patients. However, conclusions cannot be made regarding the optimal embolic material to use, because the choice is often determined by the discretion of the treating interventional radiologist on the basis of multiple factors, such as those related to the patient (vascular anatomy, target vessel, etc.) and the experience and familiarity of the interventional radiologist with the various material. 

Our study reported a technical success rate of 100% (43 out of 43) and a clinical success rate of 77% (33 out of 43). Therefore, 16 patients had evidence of re-bleeding within a 96-h interval, and they were all retreated with a new TAE procedure. The success rate of the secondary TAE treatment was 94%, since one of those 16 patients had a new episode of bleeding and needed further intervention. 

Overall, our results are consistent with those found in other published studies, where technical success rates range from 96% to 100% and clinical success rates range from 65% to 93% [2,12,19].

Furthermore, our comparison for technical and clinical success rates between patients with AWH treated with targeted TAE and those treated with blind TAE showed no statistically significant differences among them. The technical success rates were 100% in both groups, and the clinical success rates were comparable (77% and 78%, respectively).

Our study has several limitations, since it is a retrospective analysis limited to a single-center experience, and the number of patients is small, especially those treated with blind embolization, and we could not assess if patients were undergoing anticoagulant or antiplatelet drugs, since these data were not available on our digital medical records or on our RIS/PACS system. Moreover, the biggest limit of this study was the absence of a control group of patients with AWH managed with conservative treatment or open surgery, since it was not possible to identify those patients from the database of our center.

## 5. Conclusions

The results obtained from our study confirm that TAE is a safe and effective option for the treatment of spontaneous abdominal wall hematomas. Pre-procedural CTA should always be performed, since it is essential to topographically localize the hematoma, study its characteristics, detect active bleeding and to plan the diagnostic angiography, which plays an essential role especially in cases where CT-Angiography did not show active bleeding, and eventually the subsequent embolization procedure.

Moreover, our results suggest that the efficacy and safety of blind embolization is comparable to non-blind, and we believe that, in lack of angiographic proof of bleeding in case of a documented hematoma at pre-procedural CT, the choice of performing a blind embolization can be reasonable and safe and that the risk–benefit ratio could be favorable, especially in patients with risk factors or deteriorated clinical conditions. 

However, further studies are necessary to prospectively compare embolization treatment to conservative management and open surgery in abdominal wall hematomas and to deepen the knowledge about the role of blind or empiric embolization in those cases where no active bleeding can be identified on DSA.

## Figures and Tables

**Table 1:** Characteristics of our study population.

Characteristic		Value
**Age**		
	mean ± SD	71 ± 12 years
	Range	87–30
**Male**		23
**Female**		20
**Pre-procedural Hemoglobin (g/dL)**		
	mean ± SD	8.51 ± 1.87
	Range	5.1–13.7
**INR**		
	mean ± SD	1.25 ± 0.26
	Range	0.94–2.21

**Table 2:** CTA and DSA findings.

Diagnostic Finding		Value *n* (%)
**Localization of AWH**		
	Anterior and/or lateral abdominal wall	15 (35%)
	Posterior abdominal wall	28 (65%)
**CT findings**		
	Active bleeding	31 (72%)
	No proof of active bleeding	12 (28%)
**Angiographic findings **		
	Direct signs active bleeding	32 (74%)
	Indirect signs of bleeding	2 (5%)
	No proof of active bleeding	9 (21%)

**Table 3:** Outcome of PTAE, embolized arteries and timing of rebleeding after TAE.

Outcome of Percutaneous Transarterial Embolization		Value *n* (%)
	Technical success rate	43 (100%)
	Clinical success rate	33 (77%)
	Major Complications	0 (0%)
	Minor Complications	1 (2%)
**Embolized arteries**		**Value *n* (%)**
	Lumbar artery	21 (31%)
	Epigastric inferior artery	17 (25%)
	Iliolumbar artery	17 (25%)
	Deep circumflex iliac artery	7 (10%)
	Hypogastric artery	2 (3%)
	Intercostal artery	2 (3%)
	Deep femoral artery	1 (1,5%)
	Phrenic artery	1 (1,5%)
**Total number of arteries embolized**		**69**
**Timing of rebleeding after TAE**		**Value *n* (%)**
	Within 24 h	5 (11.6%)
	Between 24 to 48 h	1 (2.3%)
	Between 48 to 72 h	2 (4.6%)
	Between 72 to 96 h	2 (4.6%)
**Total of patients rebleeding during 96-h follow-up**		**10 (23%)**

**Table 4:** Comparative results of blind and targeted embolization procedures.

	Blind Embolization	Targeted Embolization	*p*-Value
Number of patients	9	34	
Male	6 (66.6%)	17 (50%)	0.6
Female	3 (33.3%)	17 (50%)	0.6
Mean age of patients (years)	68.4	71.4	0.52
Mean Pre-procedural Hemoglobin (g/dL)	8.25	8.57	0.69
Men Pre-procedural INR	1.23	1.25	0.85
Anterior AWH	4 (44%)	11 (32%)	0.77
Posterior AWH	5 (56%)	23 (68%)	0.77
Mean CT-DSA delay (hours)	6.85	8.21	0.56
Technical success rate	9 (100%)	34 (100%)	-
Clinical success rate	7 (78%)	26 (77%)	0.71
Complications rate	0 (0%)	1 (3%)	0.46

## Data Availability

Not applicable.

## References

[1] Shikhman A., Tuma F. (2020). Abdominal Hematoma.

[2] Klausenitz C., Kuehn J.-P., Noeckler K., Radosa C., Hoffmann R.-T., Teichgraeber U., Mensel B. (2020). Efficacy of transarterial embolisation in patients with life-threatening spontaneous retroperitoneal haematoma. Clin. Radiol..

[3] Hatjipetrou A., Anyfantakis D., Kastanakis M. (2015). Rectus sheath hematoma: A review of the literature. Int. J. Surg..

[4] Pode D., Caine M. (1992). Spontaneous retroperitoneal hemorrhage. J. Urol..

[5] Matalon S.A., Askari R., Gates J.D., Patel K., Sodickson A., Khurana B. (2017). Don’t Forget the Abdominal Wall: Imaging Spectrum of Abdominal Wall Injuries after Nonpenetrating Trauma. Radiographics.

[6] Dohan A., Darnige L., Sapoval M., Pellerin O. (2015). Spontaneous soft tissue hematomas. Diagn. Interv. Imaging.

[7] Buffone A., Basile G., Costanzo M., Veroux M., Terranova L., Basile A., Okatyeva V., Cannizzaro M.T. (2015). Management of patients with rectus sheath hematoma: Personal experience. J. Formos. Med. Assoc..

[8] Pierro A., Cilla S., Modugno P., Centritto E.M., De Filippo C.M., Sallustio G. (2018). Spontaneous rectus sheath hematoma: The utility of CT angiography. Radiol. Case Rep..

[9] Isokangas J.M., Perälä J.M. (2004). Endovascular embolization of spontaneous retroperitoneal hemorrhage secondary to anticoagulant treatment. Cardiovasc. Intervent. Radiol..

[10] Berná J.D., Zuazu I., Madrigal M., García-Medina V., Fernández C., Guirado F. (2000). Conservative treatment of large rectus sheath hematoma in patients undergoing anticoagulant therapy. Abdom. Imaging.

[11] Albuquerque T.V.C., Monsignore L.M., De Castro-Afonso L.H., Elias-Junior J., Muglia V.F., Abud D.G. (2020). Transarterial embolization with n-butyl cyanoacrylate for the treatment of abdominal wall hemorrhage. Diagn. Interv. Radiol..

[12] Touma L., Cohen S., Cassinotto C., Reinhold C., Barkun A., Tran V.T., Banon O., Valenti D., Gallix B., Dohan A. (2019). Transcatheter Arterial Embolization of Spontaneous Soft Tissue Hematomas: A Systematic Review. Cardiovasc. Interv. Radiol..

[13] Loffroy R.F., Abualsaud B.A., Lin M.D., Rao P.P. (2011). Recent advances in endovascular techniques for management of acute nonvariceal upper gastrointestinal bleeding. World J. Gastrointest. Surg..

[14] Sacks D., McClenny T.E., Cardella J.F., Lewis C.A. (2003). Society of Interventional Radiology Clinical Practice Guidelines. J. Vasc. Interv. Radiol..

[15] Klingler P.J., Wetscher G., Glaser K., Tschmelitsch J., Schmid T., Hinder R.A. (1999). The use of ultrasound to differentiate rectus sheath hematoma from other acute abdominal disorders. Surg. Endosc..

[16] Saad Z., Ahmed B., Mostafa R., Hicham B., Lahcen B. (2017). Conservative treatment of a psoas hematoma revealed by a lower limb palsy. Pan Afr. Med. J..

[17] Seo J.G., Yang J.C., Kim T.W., Park K.H. (2019). Intramuscular hematoma on the psoas muscle. Korean J. Neurotrauma.

[18] Baekgaard J.S., Eskesen T.G., Lee J.M., Yeh D.D., Kaafarani H.M.A., Fagenholz P.J., Avery L., Saillant N., King D.R., Velmahos G.C. (2019). Spontaneous Retroperitoneal and Rectus Sheath Hemorrhage—Management, Risk Factors and Outcomes. World J. Surg..

[19] Barral M., Pellerin O., Tran V.T., Gallix B., Boucher L.-M., Valenti D., Sapoval M., Soyer P., Dohan A. (2019). Predictors of Mortality from Spontaneous Soft-Tissue Hematomas in a Large Multicenter Cohort Who Underwent Percutaneous Transarterial Embolization. Radiology.

[20] Rimola J., Perendreu J., Falcó J., Fortuño J.R., Massuet A., Branera J. (2007). Percutaneous arterial embolization in the management of rectus sheath hematoma. Am. J. Roentgenol..

